# Clinical Validation of an Ultra High-Throughput Spiral Microfluidics for the Detection and Enrichment of Viable Circulating Tumor Cells

**DOI:** 10.1371/journal.pone.0099409

**Published:** 2014-07-07

**Authors:** Bee Luan Khoo, Majid Ebrahimi Warkiani, Daniel Shao-Weng Tan, Ali Asgar S. Bhagat, Darryl Irwin, Dawn Pingxi Lau, Alvin S. T. Lim, Kiat Hon Lim, Sai Sakktee Krisna, Wan-Teck Lim, Yoon Sim Yap, Soo Chin Lee, Ross A. Soo, Jongyoon Han, Chwee Teck Lim

**Affiliations:** 1 Mechanobiology Institute, National University of Singapore, Singapore, Singapore; 2 BioSystems and Micromechanics (BioSyM) IRG, Singapore-MIT Alliance for Research and Technology (SMART) Centre, Singapore, Singapore; 3 Department of Biomedical Engineering, National University of Singapore, Singapore, Singapore; 4 Department of Medical Oncology, National Cancer Centre Singapore, Singapore, Singapore; 5 Cancer Therapeutics Research Laboratory, National Cancer Centre Singapore, Singapore, Singapore; 6 Clearbridge BioMedics Pte Ltd, Singapore, Singapore; 7 Sequenom Inc, San Diego, California, United States of America; 8 Department of Pathology, Singapore General Hospital, Singapore, Singapore; 9 Department of Hematology-Oncology, National University Hospital, Singapore, Singapore; 10 Department of Electrical Engineering and Computer Science, Department of Biological Engineering, Massachusetts Institute of Technology, Cambridge, Massachusetts, United States of America; Institute of Molecular and Cell Biology, Biopolis, United States of America

## Abstract

**Background:**

Circulating tumor cells (CTCs) are cancer cells that can be isolated via liquid biopsy from blood and can be phenotypically and genetically characterized to provide critical information for guiding cancer treatment. Current analysis of CTCs is hindered by the throughput, selectivity and specificity of devices or assays used in CTC detection and isolation.

**Methodology/Principal Findings:**

Here, we enriched and characterized putative CTCs from blood samples of patients with both advanced stage metastatic breast and lung cancers using a novel multiplexed spiral microfluidic chip. This system detected putative CTCs under high sensitivity (100%, n = 56) (Breast cancer samples: 12–1275 CTCs/ml; Lung cancer samples: 10–1535 CTCs/ml) rapidly from clinically relevant blood volumes (7.5 ml under 5 min). Blood samples were completely separated into plasma, CTCs and PBMCs components and each fraction were characterized with immunophenotyping (Pan-cytokeratin/CD45, CD44/CD24, EpCAM), fluorescence in-situ hybridization (FISH) (EML4-ALK) or targeted somatic mutation analysis. We used an ultra-sensitive mass spectrometry based system to highlight the presence of an EGFR-activating mutation in both isolated CTCs and plasma cell-free DNA (cf-DNA), and demonstrate concordance with the original tumor-biopsy samples.

**Conclusions/Significance:**

We have clinically validated our multiplexed microfluidic chip for the ultra high-throughput, low-cost and label-free enrichment of CTCs. Retrieved cells were unlabeled and viable, enabling potential propagation and real-time downstream analysis using next generation sequencing (NGS) or proteomic analysis.

## Introduction

Circulating tumor cells (CTCs) is a collective term to describe cancer cells of solid tumor origin found in the blood of cancer patients. The heterogeneous nature of CTCs provides a comprehensive yet non-invasive means for characterizing tumor molecular subtypes, which can be utilized for stratifying patients to appropriate cancer therapy [1], [2]. Current CTC capture platforms employ flow cytometry [3], fluorescence and magnetic-activated cell sorting methods [4], gradient centrifugation [5], and filtration [6], [7], [8]. These techniques are often limited by lengthy and complicated processing procedures, low purity and cell viability. An assay with high throughput, selectivity and specificity for CTC detection is pivotal for advancing CTC characterization and utility [9]. At present, Epithelial Cell Adhesion Molecule (EpCAM) is the most popular epithelial biomarker commonly used in the detection of CTCs [10]. However, EpCAM may not be expressed in all CTCs due to epithelial-mesenchymal transition (EMT) [11], [12]. There is also growing interest in plasma cell-free DNA (cf-DNA) as an alternative for a non-invasive biomarker. Initial investigations suggest a degree of concordance between cf-DNA, CTCs [13], and disseminated tumor cells (DTCs) in metastatic breast cancer patients, highlighting the possibility that cf-DNA can be of prognostic value [11].

We previously developed a novel integrated spiral microfluidic device for CTC enrichment from whole blood [14]. Here, we adopted an enhanced and high-throughput multiplexed version that demonstrated high sensitivity by the consistent detection of viable putative CTCs (Breast cancer samples: 12–1,275 CTCs/ml; Lung cancer samples: 10–1,535 CTCs/ml) from 100% of patients' blood samples (n = 56) of clinically relevant volumes (7.5 ml). Blood samples were completely fractionated to plasma, CTCs and PBMCs components for further downstream analysis such as immunostaining (Pan-cytokeratin+/CD45-), fluorescence in-situ hybridization (FISH) (EML4-ALK) or targeted somatic mutation analysis. We also demonstrated the rare presence of EGFR-activating mutation in isolated CTC-DNA and cf-DNA, as well as original tumor-biopsy samples via targeted somatic mutation. Retrieved cells were unlabeled and hence more viable for propagation and other informative downstream analysis such as next generation sequencing (NGS) and proteomic analysis.

## Materials and Methods

### Ethics statement and clinical sample preparation

**Figure 1:**
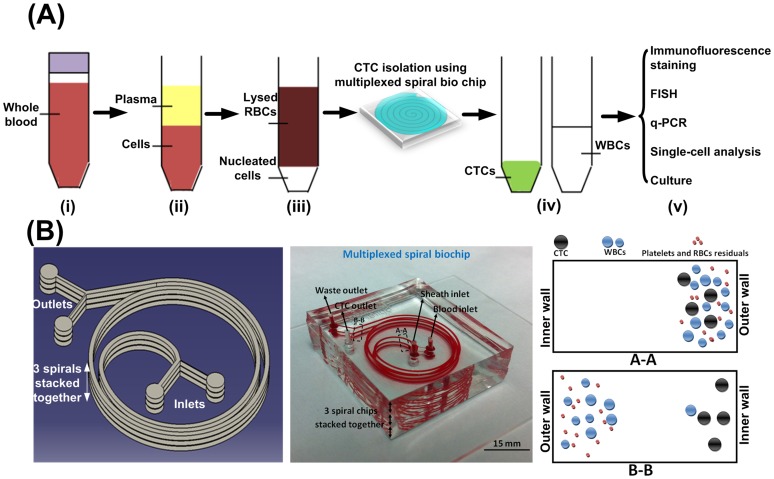
Overview of sample preparation and processing procedures. (A) Sample processing workflow showing different steps of enrichment and identification. (i) The blood sample is collected; (ii) Plasma is separated using standard centrifugation (1500×g for 10 min) and stored at −80 degree Celsius for DNA analysis. (iii) RBCs are lysed using ammonium chloride and (iv) sample is processed through multiplexed spiral chip within 10 min. (v) The isolated CTCs are available for immunostaining using standard markers or FISH (fluorescence in situ hybridization). DNA or RNA can be extracted from the CTCs and subjected to next-generation sequencing and q-PCR. Viable cells can be released and propagated in cell culture for various applications including cancer stem cell (CSC) study or drug discovery. (B) Illustration of the design of a multiplexed device (left) and optical image of an actual multiplexed spiral microfluidic device (middle) for capturing CTCs with two inlets and two outlets. Blood sample and sheath fluid are pumped through the device using two separate syringe pumps. Under the influence of inertial lift and Dean drag forces in the fluid flow, CTCs focus near microchannel inner wall (Region A-A) while WBCs and Platelets goes through one Dean cycle and migrate back towards the outer wall (Region B-B), thus achieving separation.

This study was approved by respective institutional review boards (IRB) and local ethics committee (National Healthcare Group (NHG)) (DSRB Reference 2012/00105, 2012/00979, 2010/00270, 2010/00691). Informed and written consent was obtained from all patients. IRB and ethics committee approval was also granted for NSCLC samples where retrospective archival specimens were retrieved (Singhealth 2010/516/B). Ten blood samples from healthy donors and 58 (56+2) blood samples from patients with metastatic lung or breast cancer were acquired. Blood samples were stored in EDTA-coated vacutainer tubes (Becton-Dickinson, Franklin Lakes, NJ, USA). Plasma was fractionated from whole blood for the lung samples by centrifugation (1500×*g*, 10 min). Blood samples were then lysed using red blood cell (RBC) lysis buffer (gBioscience, USA) according to manufacturer's recommendations. The nucleated cell fraction was resuspended with phosphate buffered saline (PBS) to desired concentration (Fig. 1A).

### Device fabrication

The microfluidic chips were fabricated using standard soft-lithography techniques in polydimethylsiloxane (PDMS) described elsewhere [14], [15]. After fabrication of individual chips, the multiplexed device was obtained by stacking three separate devices together using manual alignment and oxygen plasma bonding. The fluidic inlets and outlets were punched into the assembly and final device obtained by bonding the whole assembly to a pre-cleaned microscopic glass slide using plasma machine.

### Sample processing

Blood samples at 2X concentration (i.e., 7.5 ml of whole blood was lysed and resuspended in 3.75 ml of PBS) was placed into a 10 ml BD Luer-Lok syringe (Becton, Dickinson and Company) and pumped into the multiplexed chip using a syringe pump (NE-1000, New Era Pump Systems Inc., USA). Sheath consisted of 0.5% BSA in PBS supplemented with 2 mM EDTA and was similarly introduced into the biochip via a separate syringe pump (PHD 2000, Harvard Apparatus, USA). Sample and sheath were introduced into the device at a fixed flow ratio of 1∶9 for optimal separation. Device was connected to syringes and collection tubes (falcon tubes; Becton, Dickinson and Company) by Tygon tubings (Spectra-teknik, USA). The enrichment process was visualised with an inverted microscope (Olympus IX71) linked to a high-speed CCD camera (Phantom v9, Vision Research Inc., USA) and operated using the Phantom Camera Control software.

### Immunofluorescence staining and enumeration

Enriched cell fractions were fixed with 4% paraformaldehyde (PFA, Sigma, USA) for 10 min at room temperature, permeabilized with 0.1% Triton X-100 (Sigma Aldrich, USA), mixed and incubated with fluorescein isothiocyanate (FITC) conjugated pan-cytokeratin antibodies, allophycocyanin (APC) conjugated CD45 antibodies (1∶100, Miltenyi Biotec Asia Pacific, Singapore) and Hoechst 33342 dye (1M, Sigma) in PBS buffer supplemented with 0.5% BSA on ice for 30 min. Other antibodies used included EpCAM (APC), CD44 (FITC) and CD24 (APC) (all 1∶100, from Miltenyi Biotec Asia Pacific, Singapore). Stained cells were concentrated and imaged with an Olympus inverted microscope (Tokyo, Japan) (Emission filters ET460/50m, ET535/50m and ET 605/70; Olympus, Tokyo, Japan) equipped with an automated stage. Enriched cells were placed within the well of a 96-well plate (Thermo Scientific, USA) and the well was automatically scanned in a 1 mm×1 mm grid format using a programmable stage and Metamorph software (California, United States). Corresponding image sets (at 40X magnification) were compared to determine presence of putative CTCs. Hoechst-positive/pan-cytokeratin-positive (CK+)/CD45-negative (CD45-) enriched cells generally (but not exclusively) with round nucleus and high nuclear to cytoplasmic ratio were considered putative CTCs. Enriched cells of some samples were also seeded onto 2D Geltrex (Invitrogen) coated substrates and incubated at 37°C and 5% CO_2_ to allow adherence of viable cells. Non-adherent cells were washed and removed gently with 1X PBS after 72 hr. Adherent cells were then stained with FITC conjugated pan-cytokeratin antibodies and APC conjugated CD45 antibodies. Some samples were also stained for 15 min on ice with potassium iodide (PI) after microfluidic processing to determine viable proportion of enriched cells.

### DNA extraction and sequencing

DNA extraction was carried out on pooled cells (QIAamp DNA Blood Minikit (Qiagen, Hilden, Germany)), plasma DNA (QIAamp circulating nucleic acid kit (Qiagen, Hilden, Germany)) and formalin-fixed paraffin embedded tumour specimens (QIAamp DNA FFPE tissue kit (Qiagen, Hilden, Germany)). Absolute number of intact copies of extracted genomic DNA was determined by Sequenom Sample ID panel (Sequenom Inc, CA, USA). Targeted somatic mutation analysis was performed by PCR amplification followed by Single Allele Base Extension Reaction (SABER) [16] and standard iPlex chemistry [17], [18]. Resultant mutant allele products were detected by mass spectrometry (Sequenom, CA, USA). PCR amplification was performed with Sequenom PCR Reagents Set (Sequenom) (95°C, 2 minutes; 45 cycles −95°C, 30 sec; 56°C for 30 sec; 72°C, 60 sec; 72°C, 5 min). Residual dNTPs were dephosphorylated (0.5 units SAP enzyme (Sequenom)), incubated (37°C, 40 min) and enzyme deactivated (5 min, 85°C) followed by single base extension (Sequenom iPLEX Pro Kit (Sequenom) (94 °C, 30 sec; 40 cycles −95°C, 5 sec; 5 internal cycles −52°C, 5 sec; 80 °C, 5 sec; 72 °C, 3 min)). PCR products were de-salted using 6 mg of ion exchange resin (Sequenom) in 16 µl HPLC water. Cleaned PCR product was spotted onto MassArray SpectroCHIPS II (Sequenom) using the MassARRAY RS1000 Nanodispenser. MALDI-TOF MS analysis was performed using the MassArray MA4.

### DNA Fluorescence in-situ hybridization (FISH)

Cell spots were prepared with Cytospin centrifuge (Thermo Scientific, USA) (600 rpm, 6 min), fixed (acetic acid/methanol) and dehydrated via ethanol series (80%, 90%, and 100%). Slides were treated with RNase (4 mg/ml) (Sigma, USA) (40 min, 37°C), washed (1X PBS/0.2% Tween 20 (Sigma, USA)), denatured (70% formamide/2X SCC; 10 min, 80°C) and quench dehydrated via ice-cold ethanol series. EML4-ALK probe (Vysis LSI ALK breakapart, Abbott, USA) hybridization was carried out under dark and humid conditions (42°C, overnight). Hybridized slides were washed with 50% formamide/2X SSC and 2X SSC at 45°C, counterstained with 4', 6-diamidino-2-phenylindole (DAPI) and sealed.

### Statistical methods

As CTC levels in patients were not normally distributed, results were presented as counts and medians with the corresponding percentages and ranges.

## Results

### Enhanced spiral microfluidic device

The microfluidic device consisted of three stacked spiral microfluidic chips with two inlets and two outlets (Fig. 1B). Suspended cells under flow within a curvilinear microchannel experience inertial lift forces coupled with the rotational Dean drag force in the fluid regime. The combination of these forces focuses the cells at certain equilibrium positions of the channel cross-section [19]. Since these forces are a function of cell size, cells of different sizes (larger CTCs and smaller hematologic cells) occupy distinct lateral positions away from the microchannel walls, and this allows for size-based separation at the outlets [14], [20].

### Enrichment of putative CTCs from patients with metastatic breast or lung cancer

**Figure 2:**
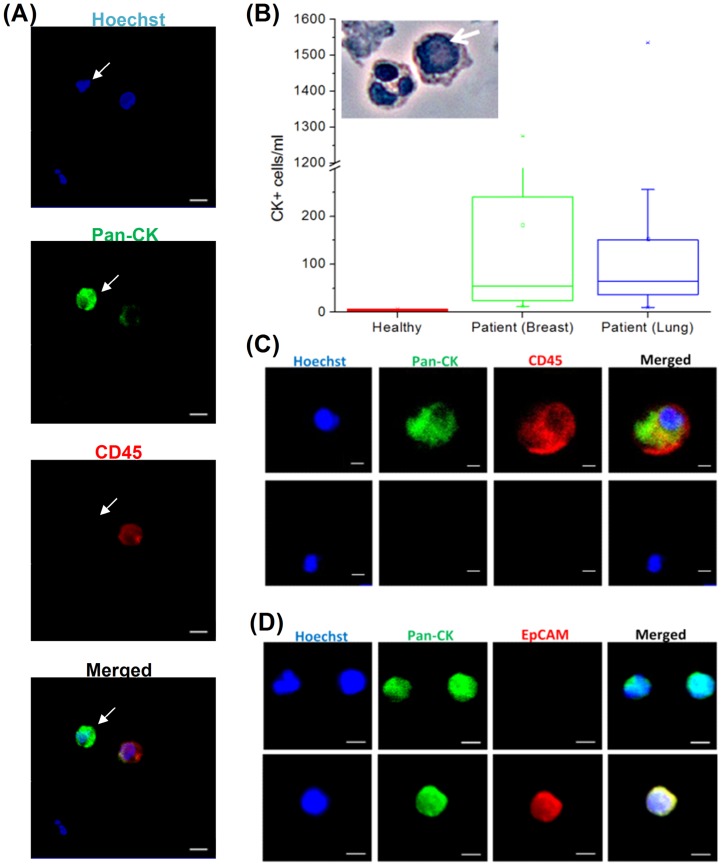
Enumeration of CTC from cancer patients. (A) Immunofluorescence staining of isolated CTCs. CTCs (marked by white arrow) were identified by the following criteria: Hoechst+, pan-CK+ and CD45-. Scale bar: 20 µm (B) Box plot summary indicating the range of CK+cells/ml recovered from the sample outlet for blood samples extracted from healthy volunteers, as wells as breast and lung cancer patients. The box plot presents the median, lower and upper quartiles (25^th^,75^th^ percentiles). Data points that lie outside the 10^th^ and 90^th^ percentiles are shown as outliers (Anova, p<0.001). Encapsulated image of PAP stained isolated cells shows a large CTC with high nucleus to cytoplasmic (N/C) ratio (labeled with white arrow). (C) Staining of CTC for pan-CK and CD45. Upper panel depicts a representative image of cells which were double positive (CK+/CD45+); while lower panel shows a double negative (CK-/CD45-) cell. Scale bar: 20 µm (D) Staining of CTC for pan-cytokeratin and EpCAM. Scale bar: 20 µm.

**Figure 3:**
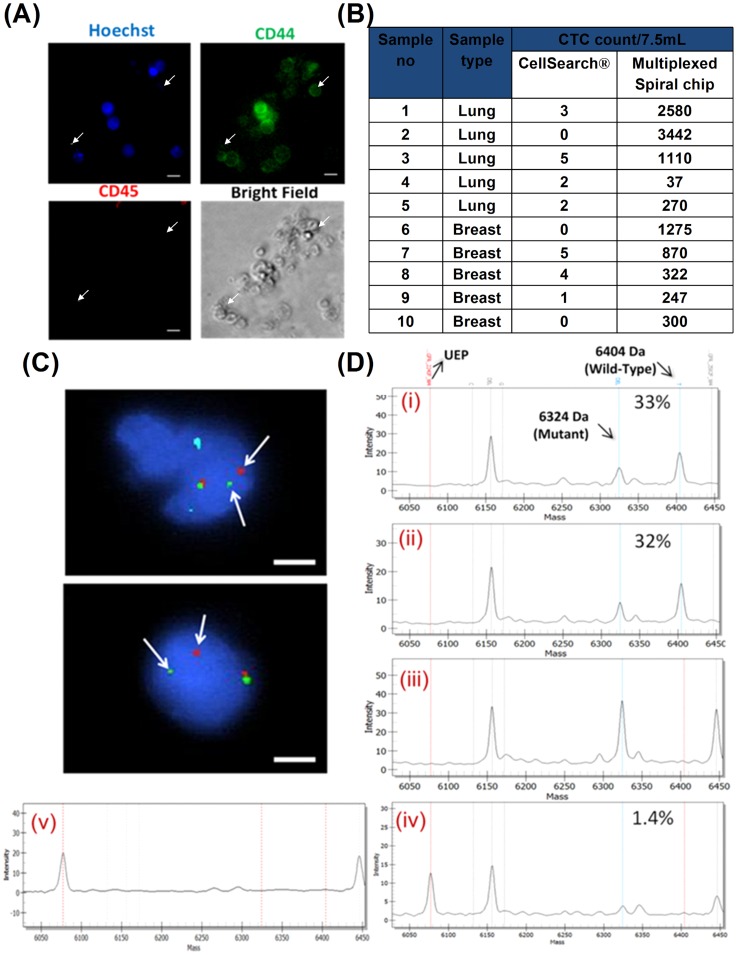
Downstream analysis of enriched CTCs. (A) CTC viability demonstrated by attachment to 2D Geltrex (Invitrogen)-coated substrate (72 hr after seeding). Isolated CTC were enriched for CD44. No cells were stained for CD45, indicating the absence of WBCs which did not adhere to substrate and were removed after washing with 1X PBS. Some CD44+cells were not stained for Hoechst (white arrows). Scale bar: 20 µm (B) Comparison of CTC isolation and recovery with CellSearch system. (C) Molecular FISH analysis on enriched CTCs of a patient with NSCLC. Cells were stained using Vysis ALK Break Apart FISH probe and counterstained with DAPI. The red and green signals demonstrated a distinct separation of the original fusion signal (arrows), indicating a rearrangement in the 2p23 ALK-gene locus. Scale bar: 16 µm. (D) MassArray spectra for a patient with NSCLC harboring EGFR L747_P753>S. Trace from FFPE, plasma and pooled CTCs illustrated. Percentage indicates calculated proportion of mutant allele against wild type allele (UEP: Unextended primer). (**i**) iPlex bi-allelic spectra on FFPE sample (33% mutant frequency), (**ii**) iPlex bi-allelic spectrum on plasma sample (32% mutant frequency), (**iii**) SABER mutant specific spectrum on plasma sample (Positive – high frequency), (**iv**) SABER mutant specific spectrum on CTCs (Positive – low frequency (n = 3/94), estimated mutant frequency of 1.4%) and (**v**) Representative iPlex & SABER (shown) spectrum on no-template control sample (Negative).

Blood samples (7.5 ml) from 10 healthy donors (Table S1 in File S1) and 58 patients (Table S2 in File S1) with metastatic breast or non-small cell lung cancer (NSCLC) were processed using the multiplexed spiral microfluidic chip. Two samples included in the table were not enumerated for CTCs and their enriched samples were directly processed for SABER molecular analysis (see section below). Hoechst positive/pan-cytokeratin-positive (CK+)/CD45-negative (CD45-) enriched cells were considered putative CTCs (Fig. 2A, 2B). These cells generally (but not exclusively) exhibit round nucleus and high nuclear to cytoplasmic ratio. CTCs were detected in 100% (n = 56) of all samples, with a varied range of CTCs isolated for breast cancer samples (12-1,275 CTCs/ml) (Median: 55 CTCs/ml) and NSCLC samples (10–1,535 CTCs/ml) (Median: 82 CTCs/ml) respectively (Fig. 2B). CK+/CD45- cells were detected at significantly lower counts in healthy samples (2–7 CK+cells/ml). These could be attributed to epithelial cells present at trace amounts in blood. However, due to their small number in comparison with that of cancer patients, a detection threshold at >7 CK+/CD45- cells was thus determined for a sample to be considered significantly positive for CTCs. Also, a negligible amount of double positive CK+/CD45+cells (<5%, data not reported) were detected in our enriched samples, as was similarly reported elsewhere [21]. However, since the nature of these cells is yet to be established, they are not considered for enumeration in our study. We also observed many Hoechst+/CK-/CD45- cells among the captured putative CTCs (Fig. 2C). This population varied in distribution across all samples, and was present at an average proportion of 51.5±17.3% of the total nucleated cells (Table S2 in File S1). Several hypothesis generated to explain their presence include the theory of cancer cell intermediates due to EMT [12], [22]. Five enriched samples were also immunostained for EpCAM, and EpCAM-/CK+ and EpCAM+/CK+ cells were detected in the isolated CTCs (Fig 2D). These EPCAM- cells constituted more than half of the enriched cell population (∼89.1±0.6%) (Fig. S1 in File S1) and are generally CD45-. A portion of CK+ putative CTCs from these 5 enriched samples were also positive for EMT markers such as E-cadherin and Vimentin (Fig. S2 in File S1). Enriched samples generally retained viability, as determined by potassium iodide staining (∼87.5%, Fig. S3 in File S1). Furthermore, a fraction of these enriched viable CTCs maintained on 2D substrates expressed CD44 (Fig. 3A), and some CD44+ cells also co-expressed CD24 (∼24.7±1.4%) (Fig. S4 in File S1). The expression of CD44 is associated with cancer stem cell-like traits [23].

### Head to head comparison with CellSearch assay

Comparisons on the CTC enumeration values between the *FDA* approved CellSearch assay and our multiplexed spiral biochip were conducted with 10 blood samples from patients with breast or lung cancer. CTCs were detected in 80% (8/10) samples using CellSearch, and 100% of the samples (10/10) by the multiplexed spiral microfluidic chip. A significantly lower range of CTC count was obtained from CellSearch as compared to the multiplexed spiral device (Fig. 3B), implying loss of EPCAM- CTCs using CellSearch. Data illustrating similar limitations in detecting lung CTCs and contrast between CTC counts obtained has been previously highlighted in comparison study between CellSearch and ISET [24].

### Identifying therapeutically tractable alterations in CTCs and plasma

Given the challenge with low tissue yield from lung biopsies, we sought to determine therapeutically tractable alterations in enriched NSCLC CTCs. EML4-ALK gene translocation is found in approximately 1 to 6.7% of NSCLC patients [25], [26]. In an index ALK positive NSCLC (sample no 18, Table S2 in File S1), we demonstrated ALK rearrangement in CTCs (Fig. 3C) using the ALK Vysis breakapart probe (Abbott Molecular, USA) after enumeration and fixation of spotted cells. Out of 177 enumerated cells, 25.4% were found to have positive signals for ALK rearrangement, with the same fusion signal identified in 54% of 200 cells in the original FFPE sample. We also performed targeted mutation profiling of both plasma and CTCs in three NSCLC patients using SABER [16], previously shown to detect rare alleles down to <0.5% frequency [18] in a single reaction. Technical replicates were performed where the amount of input template, as determined by Sample ID panel, was <150 intact template copies, such that a single mutant strand would be observed by the SABER method. Up to 128 technical replicates were performed, depending on the amount of intact extracted DNA template isolated from each sample. In all three samples, EGFR mutations were detected in diagnostic tumour specimens using the Sequenom massarray using standard iPlex chemistry. One baseline sample (sample no 32, Table S2 in File S1) demonstrated concordance across formalin-fixed paraffin-embedded tumor (FFPE) tumor block, plasma and CTCs, although at differing mutant allele frequency (33%, 32% and 1.5% respectively, Fig. 3D). Interestingly, one patient was sampled serially and showed no mutations in plasma and CTCs after treatment with gefitinib (sample no 33, Table S2 in File S1), an EGFR TKI inhibitor. In the last sample (sample no 11, Table S2 in File S1), while no mutation was detected in the circulating plasma DNA, it was detected at very low concentrations in pooled CTCs (0.05%) (TIL).

## Discussion

Progression in CTC characterization critically hinges on the development of techniques to enrich CTCs under high concentrations and purity [27]. The development of label-free and high throughput assays to obtain reliable ‘real-time’ analysis of the disease status is necessary to facilitate personalized treatment strategies [28]. Previously, we demonstrated a novel spiral microfluidics technique for the detection and enrichment of CTCs. The multiplexed version presented here had been further enhanced to provide a device of high throughput (20 times faster) (7.5 ml in less than 5 min), high sensitivity (100% detection) (3–1,535 CTCs) and selectivity (Mean: 750 WBCs/ml). Isolated CTCs remained viable and can be potentially propagated in culture.

Blood samples can be completely fractionated to plasma, CTCs and PBMCs components, which provides the opportunity to interrogate each component with genomic and transcriptomic tools. We obtained high definition images of immunostained putative CTCs (Hoechst+/pan-cytokeratin+/CD45-) and further identified therapeutically tractable genomic alterations (EML4-ALK translocation) in CTCs, using gold standard FISH assays as well as a mass spectrometry based method for mutational profiling. In a patient with paired CTCs and plasma, we demonstrated concordance in EGFR mutation in both cf-DNA and CTCs. Despite the low WBC count through the use of the spiral microfluidic biochips, somatic mutations were found in pooled CTCs at very low frequencies (1.5% and 0.05%). Possible reasons include the presence of heterogeneous cell populations in circulation whose molecular profiles are discordant from the primary. Given the amount of DNA required for this high sensitivity assay, multiplexed mutational analysis may be feasible to reliably obtain genetic patterns of CTCs. The fast processing time and label-free nature of the spiral microfluidic biochip lends itself to a broad range of potential genomic and transcriptomic applications. There are currently ongoing studies to apply RNA-based single-cell molecular analysis and next generation sequencing (NGS) on captured CTCs. The improvisation of such upcoming techniques for CTC enrichment and characterisation will hopefully shed new light on the CTC biology (origin, progression) and utilisation for therapeutics and treatment.

## Supporting Information

File S1 Contains the following Supporting Information files: **Table S1**: List of healthy samples as controls. **Table S2**: List of patient samples for clinical validation. Clinico-pathological characteristics are provided for patients with metastatic lung or breast cancer who provided samples for CTC enumeration. Samples may be serially obtained from a single patient and these are indicated by the patient number. C: Cycle, D: Day. Post sutent pre AC samples are stated to be <3 weeks post-treatment. **Figure S1**: EpCAM staining of enriched cell populations. (A) Immunostaining with EpCAM-FITC and CD45-APC antibodies. (B) Flow cytometry analysis of EpCAM/CD45 cell populations. Scale bar: 20 µm. **Figure S2**: CTC images displaying variation in EMT biomarker expression. (A) CK+ cells can either be E-cadherin+ or E-cadherin- on breast CTCs. (B) CK+ cells can either be Vimentin+ or Vimentin- on breast CTCs. Scale bar: 20 µm. **Figure S3**: Scattered plot obtained with flow cytometry analysis. Potassium iodide staining of enriched samples to determine viability. **Figure S4**: Flow cytometry analysis of CD44-FITC/CD24-APC cell populations.
(DOC)
