# Supplementary material for: Clinical Validation of an Ultra High-Throughput Spiral Microfluidics for the Detection and Enrichment of Viable Circulating Tumor Cells
Source: PLoS One. 2014 Jul 7;9(7):e99409. doi: 10.1371/journal.pone.0099409 (PMC4085042; doi:10.1371/journal.pone.0099409)

**Clinical validation of an ultra high-throughput microfluidic device for detection and enrichment of rare circulating tumor cells in blood**

Bee Luan Khoo, Majid Ebrahimi Warkiani, Daniel Shao-Weng Tan, Ali Asgar S. Bhagat, Darryl Irwin, [Dawn Lau Pingxi](https://exchange.nus.edu.sg/owa/?ae=Item&t=IPM.Note&a=New&to=dawn.lau.p.x@nccs.com.sg&nm=Dawn+Lau+Pingxi+(NCCS)), Alvin S.T. Lim, Kiat Hon Lim, [Sai Sakktee Krisna](https://exchange.nus.edu.sg/owa/?ae=Item&t=IPM.Note&a=New&to=sai.sakktee.krisna@nccs.com.sg&nm=Sai+Sakktee+Krisna+(NCCS)), Wan-Teck Lim, Yoon Sim Yap, Soo Chin Lee, Ross A. Soo, Jongyoon Han, Chwee Teck Lim

Supplementary information

**Table S1:** List of healthy samples as controls.

| **Sample no** | **Volume processed/ml** | **Pan-CK+/ml** |
| --- | --- | --- |
| **1** | 7.5 | 4 |
| **2** | 7.5 | 4 |
| **3** | 7.5 | 3 |
| **4** | 7.5 | 3 |
| **5** | 7.5 | 4 |
| **6** | 7.5 | 2 |
| **7** | 7.5 | 6 |
| **8** | 7.5 | 7 |
| **9** | 7.5 | 6 |
| **10** | 7.5 | 6 |

Table S2: List of patient samples for clinical validation. Clinico-pathological characteristics are provided for patients with metastatic lung or breast cancer who provided samples for CTC enumeration. Samples may be serially obtained from a single patient and these are indicated by the patient number. C: Cycle, D: Day. Post sutent pre AC samples are stated to be <3 weeks post-treatment.

| **Sample no** | **Patient no** | **Cancer type** | **Cancer stage** | **Treatment time point** | **Known mutation** | **CTC (Pan-CK+/CD45-) /mL** | **WBC (Pan-CK-/CD45+) /ml** | **Double negative (Pan-CK-/CD45-) cells/ml** | **Total nucleated cells/ml** |
| --- | --- | --- | --- | --- | --- | --- | --- | --- | --- |
| **1** | 1 | Lung | IV | Single draw |  | 97 | 4469 | 6413 | 10882 |
| **2** | 2 | Lung | IV | Single draw |  | 74 | 3750 | 5962 | 9712 |
| **3** | 3 | Lung | III | Baseline |  | 153 | 3993 | 7341 | 12,555 |
| **4** | 4 | Lung | IV | 2 weeks/ C1D15 |  | 151 | 2375 | 946 | 4832 |
| **5** | 5 | Lung | IIIB | Single draw |  | 459 | 9492 | 11383 | 23,172 |
| **6** | 6 | Lung | IV | Single draw |  | 33 | 308 | 616 | 1,289 |
| **7** | 7 | Lung | IV | Single draw |  | 12 | 184 | 629 | 922 |
| **8** | 8 | Lung | IV | Single draw |  | 37 | 1792 | 1726 | 3,889 |
| **9** | 9 | Lung | IIIB | Single draw |  | 30 | 15 | 574 | 885 |
| **10** | 10 | Lung | IV | Single draw |  | 135 | 29824 | 7029 | 38,469 |
| **11** | 11 | Lung | IVB | Single draw | EGFR+ | 99 | 759 | 2764 | 4,511 |
| **12** | 12 | Lung | IV | Single draw |  | 20 | 2521 | 2354 | 5023 |
| **13** | 13 | Lung | IV | Single draw |  | 256 | 10612 | 2385 | 13,766 |
| **14** | 14 | Lung | IV | Single draw |  | 90 | 399 | 4964 | 6,258 |
| **15** | 15 | Lung | IV | Single draw |  | 344 | 11033 | 14476 | 28,951 |
| **16** | 16 | Lung | IV | Single draw |  | 43 | 213 | 613 | 1,298 |
| **17** | 17 | Lung | IIIB | Single draw |  | 148 | 1508 | 7337 | 10,321 |
| **18** | 18 | Lung | IV | Single draw | ALK positive | 1535 | 3649 | 3845 | 16148 |
| **19** | 19 | Lung | IIIB | Single draw |  | 13 | 4234 | 6234 | 10468 |
| **20** | 20 | Lung | IV | Single draw |  | 459 | 3139 | 5142 | 8281 |
| **21** | 21 | Lung | IV | Baseline |  | 52 | 16510 | 14354 | 30864 |
| **22** | 22 | Lung | IV | Baseline |  | 35 | 25854 | 23256 | 49110 |
| **23** | 22 | Lung | IV | 2 weeks/C1D1 |  | 10 | 3769 | 7420 | 11237 |
| **24** | 22 | Lung | IV | 4 weeks/ C2D1 |  | 43 | 7283 | 6250 | 13967 |
| **25** | 23 | Lung | IV | 4 weeks/ C2D1 |  | 46 | 6401 | 7276 | 13905 |
| **26** | 24 | Lung | IV | 2 weeks/C1D1 |  | 127 | 5984 | 5053 | 12308 |
| **27** | 24 | Lung | IV | 2 weeks/C1D8 |  | 160 | 11953 | 1826 | 15378 |
| **28** | 24 | Lung | IV | 2 weeks/C1D15 |  | 59 | 3353 | 2430 | 6368 |
| **29** | 25 | Lung | IV | Single draw |  | 153 | 3993 | 7341 | 12555 |
| **30** | 25 | Lung | IIIB | 4 weeks/C1D15 |  | 20 | 11223 | 10821 | 22044 |
| **31** | 25 | Lung | IV | 4 weeks/ C2D1 |  | 65 | 16191 | 12891 | 29082 |
| **32** | 26 | Lung | IV | Single draw | EGFR+ | NA | NA | NA | NA |
| **33** | 27 | Lung | IV | Single draw | EGFR+ | NA | NA | NA | NA |
| **34** | 28 | Lung | IV | 2 weeks/C1D1 |  | 42 | 4430 | 4856 | 9494 |
| **35** | 28 | Lung | IV | 4 weeks/ C2D1 |  | 46 | 6629 | 7276 | 13905 |
| **36** | 29 | Breast | IV | Baseline |  | 34 | 2133 | 2122 | 4456 |
| **37** | 30 | Breast | IV | Baseline |  | 67 | 6118 | 6716 | 13232 |
| **38** | 30 | Breast | IV | 2 weeks/C1D1 |  | 99 | 681 | 1364 | 2,785 |
| **39** | 31 | Breast | IV | Baseline |  | 12 | 1634 | 4813 | 6535 |
| **40** | 32 | Breast | IV | Baseline |  | 56 | 5045 | 4864 | 10327 |
| **41** | 32 | Breast | IV | Post Sutent |  | 20 | 5920 | 8067 | 14135 |
| **42** | 32 | Breast | IV | 2 weeks/C1D15 |  | 20 | 17237 | 4137 | 21521 |
| **43** | 33 | Breast | IV | Baseline |  | 15 | 1093 | 3207 | 4410 |
| **44** | 33 | Breast | IV | Post Sutent |  | 17 | 1596 | 1239 | 2919 |
| **45** | 34 | Breast | IV | Baseline |  | 24 | 6775 | 2326 | 9279 |
| **46** | 34 | Breast | IV | Post Sutent |  | 44 | 2078 | 2627 | 5035 |
| **47** | 35 | Breast | IV | Post Sutent |  | 61 | 17641 | 11844 | 29939 |
| **48** | 35 | Breast | IV | 2 weeks/C1D15 |  | 55 | 9 | 1715 | 2133 |
| **49** | 36 | Breast | IV | 2^nd^ tumor assessment |  | 1275 | 4861 | 6873 | 11734 |
| **50** | 37 | Breast | IV | Pre-treatment |  | 870 | 4068 | 6081 | 10149 |
| **51** | 38 | Breast | IV | Progression |  | 322 | 4185 | 6297 | 10482 |
| **52** | 39 | Breast | IIIB | 2^nd^ tumor assessment |  | 247 | 3695 | 5766 | 9461 |
| **53** | 40 | Breast | IV | 2^nd^ tumor assessment |  | 300 | 3813 | 5639 | 9452 |
| **54** | 41 | Breast | IV | Baseline |  | 76 | 1705 | 2873 | 5,146 |
| **55** | 42 | Breast | IV | Single draw |  | 120 | 415 | 1372 | 2,716 |
| **56** | 43 | Breast | IV | Single draw |  | 52 | 4986 | 5186 | 4596 |
| **57** | 44 | Breast | IIIA | 2 weeks/C1D1 |  | 240 | 15308 | 13106 | 28414 |
| **58** | 45 | Breast | IV | Baseline |  | 40 | 11845 | 11664 | 23910 |

**Figure S1**: EpCAM staining of enriched cell populations. (A) Immunostaining with EpCAM-FITC and CD45-APC antibodies. (B) Flow cytometry analysis of EpCAM/CD45 cell populations. Scale bar: 20 µm


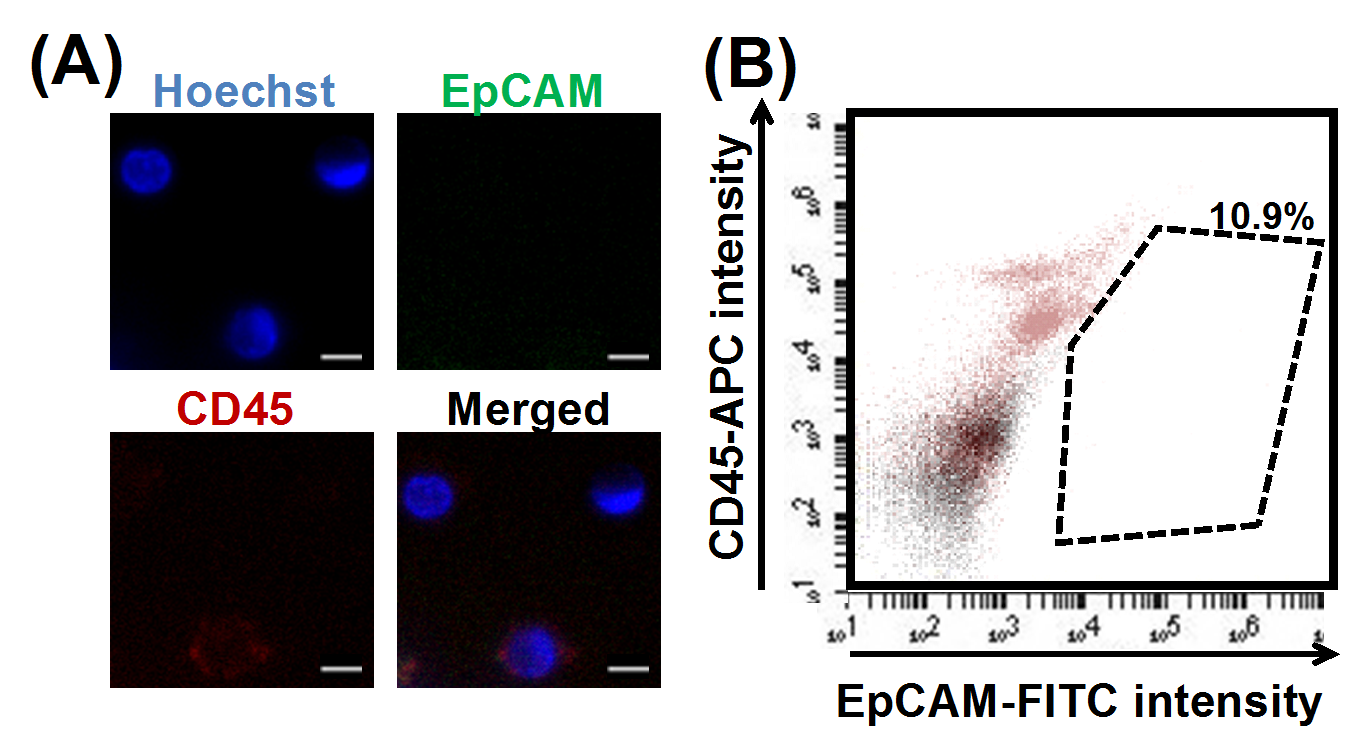


**Figure S2**: CTC images displaying variation in EMT biomarker expression. (A) CK+ cells can either be E-cadherin+ or E-cadherin- on breast CTCs. (B) CK+ cells can either be Vimentin+ or Vimentin- on breast CTCs. Scale bar: 20 µm


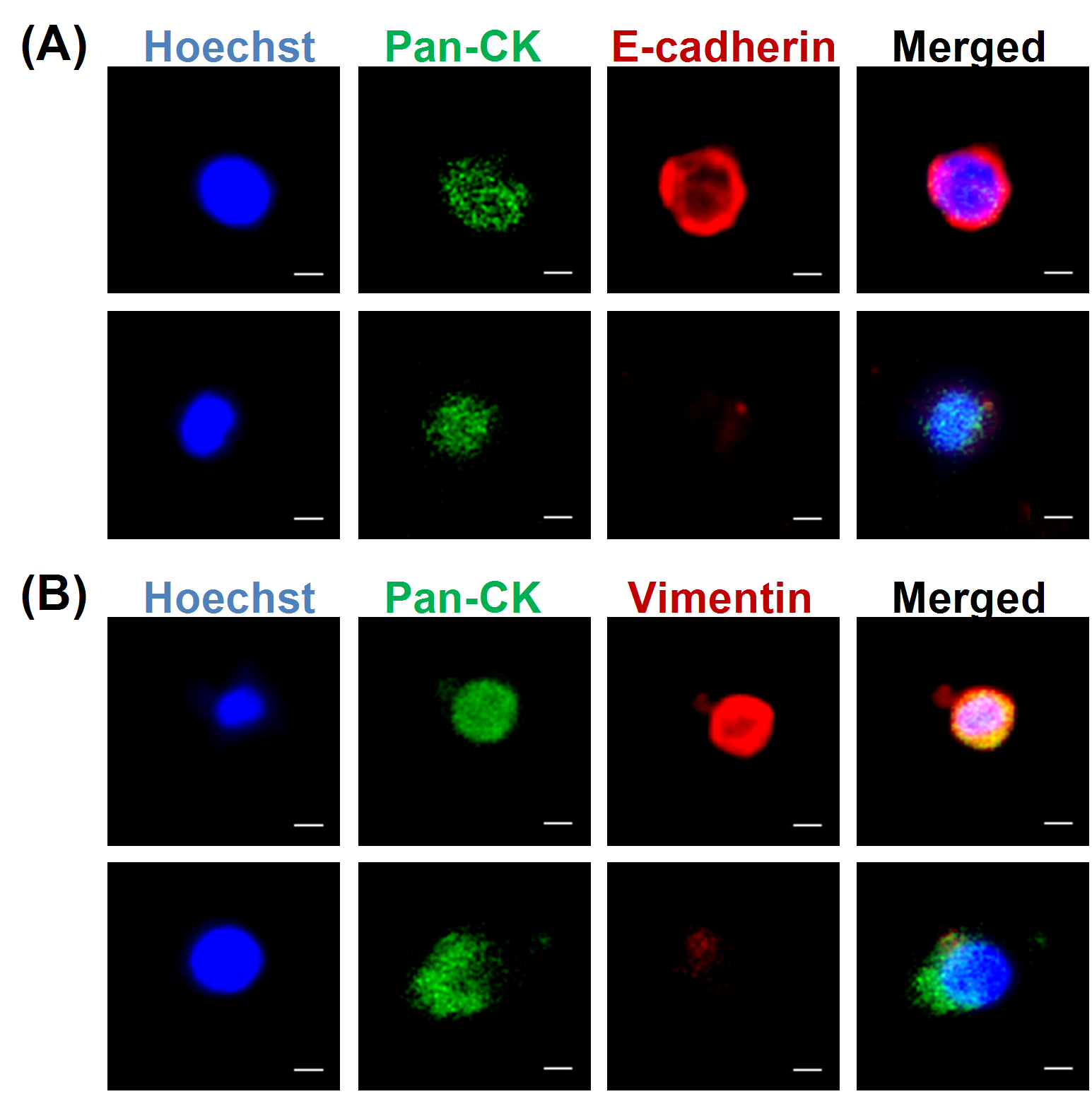


**Figure S3**: Scattered plot obtained with flow cytometry analysis. Potassium iodide staining of enriched samples to determine viability.

**
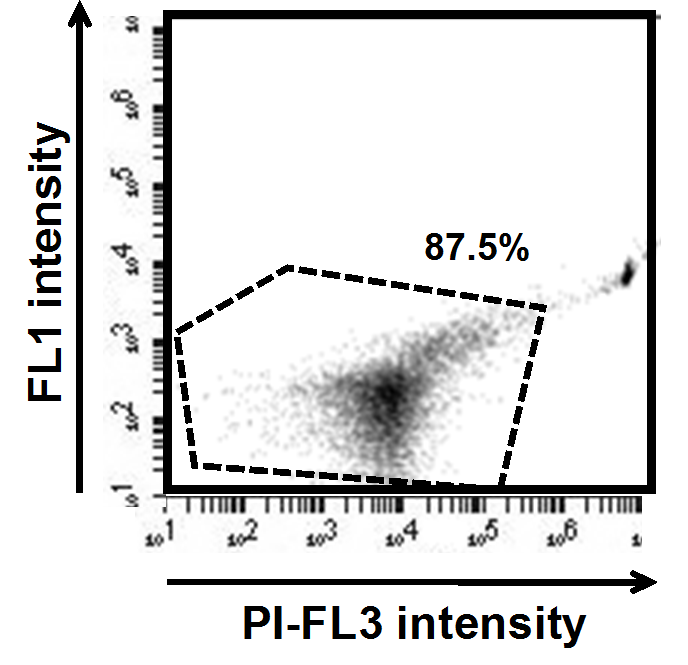
**

**Figure S4**: Flow cytometry analysis of CD44-FITC/CD24-APC cell populations.


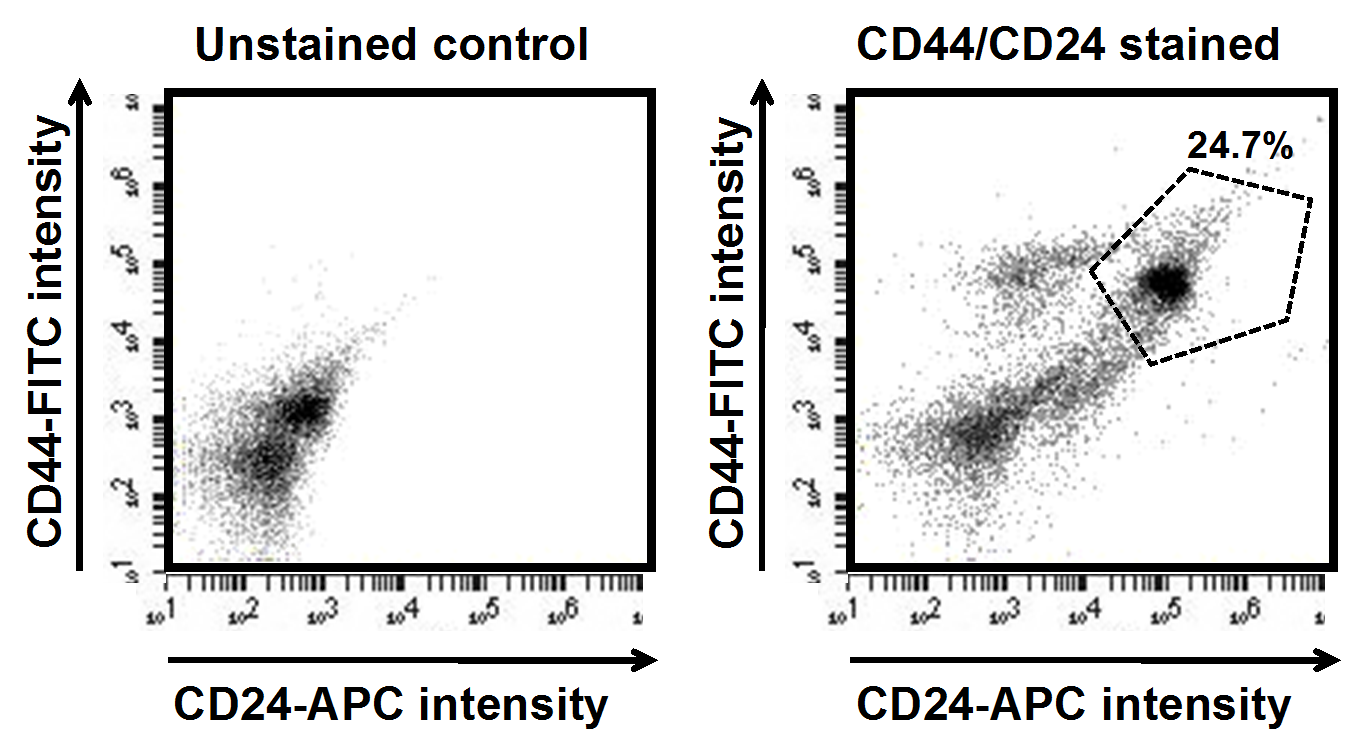

Supplement: File S1 — Contains the following Supporting Information files: Table S1: List of healthy samples as controls. Table S2: List of patient samples for clinical validation. Clinico-pathological characteristics are provided for patients with metastatic lung or breast cancer who provided samples for CTC enumeration. Samples may be serially obtained from a single patient and these are indicated by the patient number. C: Cycle, D: Day. Post sutent pre AC samples are stated to be <3 weeks post-treatment. Figure S1: EpCAM staining of enriched cell populations. (A) Immunostaining with EpCAM-FITC and CD45-APC antibodies. (B) Flow cytometry analysis of EpCAM/CD45 cell populations. Scale bar: 20 µm. Figure S2: CTC images displaying variation in EMT biomarker expression. (A) CK+ cells can either be E-cadherin+ or E-cadherin- on breast CTCs. (B) CK+ cells can either be Vimentin+ or Vimentin- on breast CTCs. Scale bar: 20 µm. Figure S3: Scattered plot obtained with flow cytometry analysis. Potassium iodide staining of enriched samples to determine viability. Figure S4: Flow cytometry analysis of CD44-FITC/CD24-APC cell populations. (DOC) [file pone.0099409.s001.doc]
