# Supplementary material for: Correction: Clinical Validation of an Ultra High-Throughput Spiral Microfluidics for the Detection and Enrichment of Viable Circulating Tumor Cells
Source: PLoS One. 2014 Oct 10;9(10):e111296. doi: 10.1371/journal.pone.0111296 (PMC4193886; doi:10.1371/journal.pone.0111296)
Supplement: Table S2 — List of patient samples for clinical validation. Clinico-pathological characteristics are provided for patients with metastatic lung or breast cancer who provided samples for CTC enumeration. Samples may be serially obtained from a single patient and these are indicated by the patient number. C: Cycle, D: Day. Post sutent pre AC samples are stated to be <3 weeks post-treatment. (DOCX) [file pone.0111296.s001.docx]

# Correction: Clinical Validation of an Ultra High-Throughput Spiral Microfluidics for the Detection and Enrichment of Viable Circulating Tumor Cells

The *PLOS ONE* Staff

There is an error in the 10^th^ sentence of the first paragraph in the Enrichment of putative CTCs from patients with metastatic breast and lung cancer section of the Results. The correct sentence is: This population varied in distribution across all samples, and was present at an average proportion of 48.8±15.5% of the total nucleated cells (Table S2 in File S1).

Table S2 in File S1 is incorrect. The headings for the last three columns are incorrectly labeled and the total nucleated cell counts are incorrect. Please view the correct Table S2 below.

## Supporting Information

**Table S2.** List of patient samples for clinical validation. Clinico-pathological characteristics are provided for patients with metastatic lung or breast cancer who provided samples for CTC enumeration. Samples may be serially obtained from a single patient and these are indicated by the patient number. C: Cycle, D: Day. Post sutent pre AC samples are stated to be <3 weeks post-treatment.

## Reference

Khoo BL, Warkiani ME, Tan DS-W, Bhagat AAS, Irwin D, et al. (2014) Clinical Validation of an Ultra High-Throughput Spiral Microfluidics for the Detection and Enrichment of Viable Circulating Tumor Cells. PLoS ONE 9(7): e99409. doi:10.1371/journal.pone.0099409
